# Changes in cerebrovascular reactivity within functional networks in older adults with long-COVID

**DOI:** 10.3389/fneur.2025.1504573

**Published:** 2025-03-26

**Authors:** Jessica M. Pommy, Alexander Cohen, Amarpreet Mahil, Laura Glass Umfleet, Sara J. Swanson, Malgorzata Franczak, Shawn Obarski, Kelly Ristow, Yang Wang

**Affiliations:** ^1^Division of Neuropsychology, Department of Neurology, The Ohio State University Wexner Medical Center, Columbus, OH, United States; ^2^Department of Radiology, Medical College of Wisconsin, Milwaukee, WI, United States; ^3^Department of Neurology, Medical College of Wisconsin, Milwaukee, WI, United States; ^4^Department of Biophysics, Medical College of Wisconsin, Milwaukee, WI, United States

**Keywords:** long-COVID, neurovascular, cerebrovascular reactivity, functional MRI, aging, brain network, individual, cognition

## Abstract

**Introduction:**

Cognitive symptoms are reported in the vast majority of individuals with long-COVID and there is growing support to suggest neurovascular mechanisms may play a role. Older adults are at increased risk for developing complications associated with COVID-19, including heightened risk for cognitive decline. Cerebrovascular Reactivity (CVR), a marker of neurovascular health, has been linked to age related cognitive decline and may play a role in long-COVID, however, this has not yet been explored.

**Methods:**

The present study examined group differences in CVR in 31 older adults with long-COVID compared to 31 cognitively unimpaired older adults without long-COVID symptoms. Follow up analyses were conducted to examine how CVR was associated with both subjective cognitive symptoms and neuropsychological (NP) test performance. A subject-specific approach, Distribution-Corrected Z-scores (DisCo-Z), was used.

**Results:**

Analyses revealed the long-COVID group demonstrated significantly greater incidence of extreme CVR clusters within the brain (>100 voxels) and within functional networks thought to drive attention and executive function. Extreme positive CVR clusters were positively associated with greater number of subjective cognitive symptoms and negatively correlated with NP performance.

**Discussion:**

These findings are among the first to provide a link between cognitive functioning in long-COVID and neurovascular changes relevant for aging and mechanistic studies of long-COVID.

## 1 Introduction

Despite significant advances in the prevention and management of acute COVID-19, a subset of individuals will continue to experience persistent symptoms after resolution of acute infection, a condition known as “long-COVID” (1–6). Long-COVID is a multi-organ disease that can affect individuals irrespective of hospitalization status, with symptoms lasting months or even years (7). Prevalence estimates vary, but the World Health Organization estimated 10–20% of individuals with acute infections will develop mild to moderate long-COVID symptoms, with prevalence estimates reaching up to 45% when different diagnostic criteria is applied (5, 8). While the clinical presentation of long-COVID is heterogenous (9), neurological symptoms are particularly prevalent among individuals that experienced mild acute infections (9–11), and are associated with declines in daily functioning and quality of life (2, 12). Approximately 80% of individuals with long-COVID report cognitive symptoms (2, 9, 10), often involving aspects of attention, memory, and word finding (13). Notably, these subjective cognitive concerns, again, do not appear to correlate with severity of acute infection (13–16). Given that subjective cognitive decline is a known risk factor for subsequent *objective* cognitive decline in older adults, this population may be especially vulnerable to cognitive impairment in the context of long-COVID. Older adults are more susceptible to both acute COVID-19 complications and the long-term effects of the virus, including cognitive decline (17–19). One study found that worsened perceived cognition (based on informant report) in older adults 6 months following acute infection, relative to an older adult control group (17). Further, increased incidence of neurodegenerative disease diagnosis has been observed in the year following COVID-19 infection (19). Cognitive decline in older adults with long-COVID could potentially reflect an unmasking of a preexisting neurodegenerative process or an exacerbation of cognitive decline observed in “normal” aging (18).

While the mechanisms driving long-COVID are complex, the presence of persistent endothelial cell dysfunction is of interest (20–23). It has been observed independent of comorbid vascular health conditions, acute infection severity, and examined demographic factors (age, sex) (20). Further, it plays a role in inflammatory and neuroimmune processes also associated with neurological symptoms in long-COVID (20–24). Cerebrovascular reactivity (CVR) is a measure of the vasculature system's responsiveness to vasoactive stimuli that is dependent upon cerebral endothelial function (25) making it of interest for long-COVID. Further, CVR may be particularly sensitive to cognitive symptoms in older adults with long-COVID because (1) CVR declines are observed in older adulthood (26–28), (2) reduced CVR has been associated with cognitive decline in normal aging, and (3) reduced CVR is observed in neurodegenerative conditions (27, 29). Further, advances in CVR methods have enabled CVR to be assessed safely using task fMRI (i.e., the breath holding task (30, 31)). Given the diffuse nature of long-COVID (32, 33), one might not necessarily expect a consistent focal change to manifest uniformly across individuals. For this reason, a subject-specific abnormalities (SSA) framework was employed. SSA was developed to address variability observed traumatic brain injury (TBI) and multiple sclerosis (MS), where the location of brain changes is expected to vary between patients (34–38). More specifically, we used distribution-corrected z-scores (DisCo-Z), which has been applied to a number of different neuroimaging methods (e.g., Diffusion Tensor Imaging, resting state functional connectivity) and enables one to examine clusters of extreme values within participants data across regions of the brain.

The present study examined the relationship between long-COVID, neurovascular health, and aspects of cognition in older adults. A subject-specific abnormalities (SSA) approach was used. Group differences in extreme CVR clusters in a sample of older adults with cognitive concerns in the context of long-COVID were examined relative to a group of cognitively unimpaired older adults. The clinical significance of group differences in CVR was then examined using objective and subjective cognitive assessments.

## 2 Methods

### 2.1 Participants

Participants (50 years of age and older) were recruited as part of two, larger neuroimaging studies of long-COVID. The long-COVID group was recruited from a multispecialty long-COVID clinic within a local hospital via clinician referral or through retrospective chart review. Given the notable heterogeneity in clinical presentation in long-COVID, our sample focused specifically on older adults that: (1) sought care in a multispecialty long-COVID clinic, (2) presented with persistent *subjective* changes in cognition that the individual attributed to prior COVID-19 infection (i.e., symptoms emerged following infection and remained at time of study enrollment), (3) had a previous diagnosis of COVID-19 verified within the medical record (i.e., positive COVID-19 test), and (4) had no exclusionary concomitant neurologic diagnosis, such as stroke, epilepsy, severe head injury. All participants studied had experienced mild acute infection (i.e., no hospitalization, supplemental oxygen). A matched control group was recruited from the community and comprised of older adults that: (1) expressed no subjective cognitive concerns, (2) were deemed cognitively normal based on neuropsychological exam, (3) reported no long-COVID symptoms, and (4) had no prior diagnosis indicative of cognitive decline. Given the widespread prevalence of COVID-19 infection, as well as the potential for asymptomatic infection, we could not objectively confirm absence of COVID-19 infection in the control group. Participants in the long-COVID cohort tested positive for COVID-19 between July 2020 and March 2023. Study participants were recruited and scanned between August 2021 and November 2023.

### 2.2 Cognitive assessment and symptom measures

Individuals within the matched control group underwent a brief standard neuropsychological testing battery comprised of test measures typically administered as part of a larger neuropsychological evaluation in the long-COVID clinic. Present analyses were limited to memory [delayed free recall from Rey Auditory Verbal Learning Test (RAVLT) or California Verbal Learning Test (CVLT) (39, 40)], letter fluency [total words across three letter fluency trials from FAS or Delis–Kaplan Executive Functioning System (D-KEDFS) (41, 42)], Category Fluency (total words for semantic fluency from COWAT or DKEFS) (41, 42), speeded visual attention (Trails A or Number Sequencing Trial from DKEFS) (42, 43), and speeded mental flexibility (Trails B or Number-Letter Sequencing Trial from DKEFS) (42, 43). Data for the similar measures described above were collapsed into a single variable and transformed to the same scale (e.g., scaled scores from DKEFS were transformed to Standard Score measurement).

Individuals within the long-COVID group completed a brief study questionnaire documenting cognitive concerns and impact on quality of life (*N* = 29). The severity, count, time-course, and onset time of all long-COVID symptoms were documented. Data obtained as part of the work up within the long-COVID clinic were collected as well. A subset of the long-COVID participants also underwent a clinical neuropsychological evaluation as part of standard clinical care. Data from neuropsychological evaluations was included in analyses when possible.

### 2.3 MRI acquisition

All scans were performed using the Nova Medical 32-Channel coil on one of two GE Healthcare Premier 3.0T MRI scanners. T1-weighted anatomical images were collected. Breath holding task fMRI was then acquired using a multiband, multi-echo (MBME) echo planar imaging (EPI) sequence with MB acceleration factor = 4 and three echoes. Additional parameters were as follows: TR/TE = 1,000/112,948 ms, 44 total slices, FOV=24 cm, 3 × 3 × 3 mm voxel size, partial Fourier factor = 0.85, and in-plane acceleration factor = 2. MNI resolution was 2 × 2 × 2 mm.

### 2.4 Cerebrovascular reactivity (CVR)

#### 2.4.1 Breath-holding task

CVR was examined using a previously established breath holding task performed by participants while in the MRI scanner (30). Holding one's breath increases the end tidal pressure carbon dioxide (a surrogate for arterial partial pressure of carbon dioxide) temporarily by reducing the respiratory elimination of carbon dioxide. When the task is performed during fMRI scan, CVR can be calculated as the ratio of cerebral blood flow (CBF) change to vasoactive stimuli. Briefly, the participant is instructed to modify his or her breathing over the course of the task. Instructions are presented on the screen to aid the participant throughout the task. Initially, the participant is instructed to perform paced breathing (66 s), followed by four cycles of paced breathing (24 s), breath holding on expiration (16 s of breath holding), and a brief period of self-paced recovery breathing (16 s). Scans ended with 30 s of paced breathing. Participants practiced the task first to demonstrate an understanding of task instructions and to ensure the task can be performed. The duration of the breath holding task was ~6 min. Furthermore, a respiratory trace was acquired to verify the subject performed the task. The reader is referred to Cohen and Wang (2019) for a more comprehensive description of the task.

#### 2.4.2 fMRI data preprocessing

First, the anatomical images were coregistered to MNI space using *flirt* (44, 45) for linear registration followed by *fnirt* (46) for non-linear registration. For the functional datasets, the first eight volumes were discarded to allow the signal to reach equilibrium, and then the first-echo dataset was volume registered to the first volume using *mcflirt* in FSL. Echoes 2 and 3 were registered using the transformation matrices from the first echo. Then, multiecho independent component analysis (MEICA) was run using tedana v0.0.12 which optimally combines the three echoes, determines non-bold components and regresses those components to denoise the data (47–50). The denoised data was registered to MNI space and the data was smoothed using a 6 mm FWHM Gaussian kernel. Data from two separate studies were combined for the present analyses. Because participants were scanned on one of two 3.0T MRIs, ComBat harmonization was used to address scanner-specific effects (51–53).

#### 2.4.3 CVR analyses

The CVR response during the breath-holding task was quantified by computing the percentage signal change during the breath-holding task in gray matter within cortical and subcortical regions. Voxel-wise analyses were performed and an independent *t*-test was used to determine the difference between the COVID-19 and healthy control groups. Group results were thresholded at *p* < 0.05 and cluster-size corrected using *3dClustSim* (54) in AFNI with α < 0.05. Minimum cluster size at *p* < 0.05 was 1,066 for α < 0.05.

CVR totals within the whole brain and within each of the seven Yeo resting-state networks (55), were examined between groups. Prior research has shown that resting-state can be reliably parcellated into seven function networks based on correlated patterns of correlated brain activity during resting state. The nomenclature used to label each of the networks [including visual, somatomotor, dorsal attention, ventral attention, limbic, frontoparietal, and default mode networks (DMN)] reflects functions typically associated with brain regions within that network.

Briefly, the DMN traditionally has been conceptualized as a task-negative network (and is comprised of the specific brain regions that are activated on fMRI when a participant is not performing a cognitive task), while the remaining six functional networks were named based on activation during a corresponding tasks (e.g., dorsal and ventral attention networks reflecting differential networks that are functionally active during attention tasks). Each of the seven Yeo resting-state networks, also characterized as Region of Interest (ROI) analyses, as the analysis is limited to the specific regions of that resting state network.

Distribution Corrected Z-scores (DisCo-Z) were calculated from CVR maps. Briefly, DisCo-Z enables one to examine whether extreme values are present within individual participant's neuroimaging data (i.e., subject-specific data points), followed by an analysis of the frequency of extreme values differs between cohorts (34). The control group was used as the reference group. Mean and standard deviation maps were computed for the control subjects. Individual z-scores maps were created for all subjects subtracting the mean CVR from individual CVR and dividing by the standard deviation.


$$\begin{array}{l} \frac{{CVR_{ind}-CVR}_{mean}}{CVR^{\sigma }} \end{array}$$

Z-score thresholds were adjusted for control and COVID groups separately based on Ref. 34 to reduce bias resulting in thresholds of 1.87 and 2.02 for control and COVID groups, respectively for alpha equal to 0.028.

The presence and size of extreme clusters of increased or decreased CVR (minimum size of 100 voxels) within the whole brain and within the seven Yeo resting-state networks was generated.

### 2.5 Statistical analysis

Group differences on demographic measures were examined using *t*-tests and chi-square. Group differences in incidence and size of extreme CVR clusters were examined using non-parametric statistics. More specifically, Mann-Whitney Tests were used to examine group differences in total number of extreme positive clusters and total number of extreme negative clusters within the whole brain, as well as number of ROIs with positive clusters and number of ROIs with negative clusters. Mean cluster size was then examined within the whole brain and within each ROI (7 Yeo networks). *P*-values were corrected for multiple comparisons using a Benjamini-Hochberg correction. Finally, the relationship between clinical variables and extreme CVR metrics was examined using Spearman's Rank Correlation. To support the utility of the DisCo-Z approach, group differences in voxel-wise CVR were examined as well.

## 3 Results

### 3.1 Sample characteristics

Thirty-one older adults with long-COVID (7 males, 24 females) and 31 cognitively unimpaired healthy older adults (8 males, 23 females) for whom CVR data were available were included in aforementioned analyses (see Table 1). As the long-COVID sample, was heavily weighted toward female (~1 male to 3 females) controls were matched by sex. The groups did not differ significantly on sex (*p* = 0.478) or years of education (*p* = 0.120). However, group differences in age were significant (*p* = 0.031), with the long-COVID group (mean age of 60.81 years) ~5 years younger than the healthy control cohort (mean age of 65.52 years). The sample was predominantly comprised of non-Hispanic, White participants, and there was no significant difference between groups on race or ethnicity. Regarding long-COVID symptoms, participants all endorsed cognitive decline following long-COVID. Participants within the long-COVID cohort were asked to rate which specific cognitive domains were impacted on a questionnaire.

**Table 1:** Sample characteristics^a^.

**Sample characteristics**	**CU**	**LC**	***p*-value^b^**	**N^c^**
Mean age in years	65.52	60.81	0.028	62
(S.D.)	(8.57)	(8.24)		
Sex (N)				
Male	8	7	0.478	62
Female	23	24		
Race (N)				
American Indian/Alaska Native	0	1		62
Asian	0	0		
Black/African American	0	0		
Hispanic/Latino	0	1		
Middle Eastern/North African	0	0		
Native Hawaiian/Pacific Islander	0	0		
White	31	29		
Education in years	16.9	15.6	0.120	45
(S.D.)	(1.8)	(2.8)		
Concerns endorsed by domain (%)			-	28
Memory		90.3		
Attention/concentration or		80.6		
“brain fog”				
Speech, language, or word		80.6		
finding				
Multitasking/problem solving		80.6		
Endorsed impact on functioning (%)		58.1		

^a^Study sample characteristics are presented for participants in both groups. As mentioned, data was combined from two studies, the latter of which collected additional data on sample characteristics. CU, cognitively unimpaired; LC, long-COVID; N, sample size; S.D., standard deviation.

^b^The significance of group comparisons on demographic variables is provided.

^c^Sample size represented for each variable.

#### 3.1.1 Group differences in cognitive measures

Neuropsychological data was available for 21 of the 31 participants in the long-COVID group and all the control participants. All neuropsychological test scores were standardized using matched normative reference groups consistent with standard clinical procedures and test manuals to control for the effect of demographic variables (i.e., age, sex, education). The long-COVID group scored significantly lower on memory relative to the control group (*p* = 0.036), however, group differences on remaining tasks were not significantly different. Please see Table 2 for *p*-values and group means across measures.

**Table 2:** Neuropsychological performance^a^.

**Neuropsychological variable**	**CU (*N* = 30)**	**LC (*N* = 21)**	***p*-value^b^**
Delayed recall	114.07 (13.70)	105.62 (13.78)	0.036
Trails A	114.1 (13.88)	109.14 (11.74)	0.188
Trails B	112.23 (13.71)	105.05 (12.75)	0.064
Letter fluency	108.03 (14.76)	102.57 (17.39)	0.233
Category fluency	103.57 (18.81)	100.14 (14.62)	0.488

^a^Scores are standardized to age and education and presented as Standard Scores with mean of 100 and standard deviation of 15. Values within the table reflect each group's mean Standard Score with the standard deviation in parentheses. S.d., standard deviation; CU, cognitively unimpaired; LC, long-COVID; N, sample size. *P*-Values reflect the group differences generated using *t*-tests.

^b^The significance of group comparisons on neuropsychological variables.

#### 3.1.2 Group differences in voxel-wise CVR

Voxel-wise CVR analyses revealed no significant differences between groups following cluster-wise correction for multiple comparison (*p* < 0.05, α < 0.05).

### 3.2 CVR and age within the full sample

Follow up analyses were conducted to examine the relationship between age and whole brain CVR measures given that the groups differed on age. Spearman Correlations between age and extreme positive CVR values were not statistically significant. Of note, age showed a significant negative correlation with extreme negative CVR values. However, the vast majority of analyses presented focused on extreme positive CVR values. See Supplemental material for correlations and *p*-values.

### 3.3 Group differences in extreme CVR clusters: whole brain analysis

**Figure 1:**
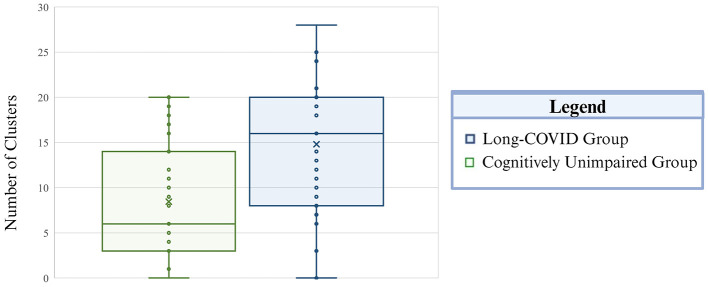
Total incidence of extreme positive CVR clusters is presented by group. The Long-COVID group is presented in blue and the cognitively unimpaired control group is depicted in green. The whiskers mark the 5th and 95th percentile, the top and bottom of the box represent the 25th and 75th percentile, respectively. The center line within each plot corresponds to the median value (50th percentile) and the “X” indicates the mean value per group. Total number of clusters is on the vertical axis.

**Figure 2:**
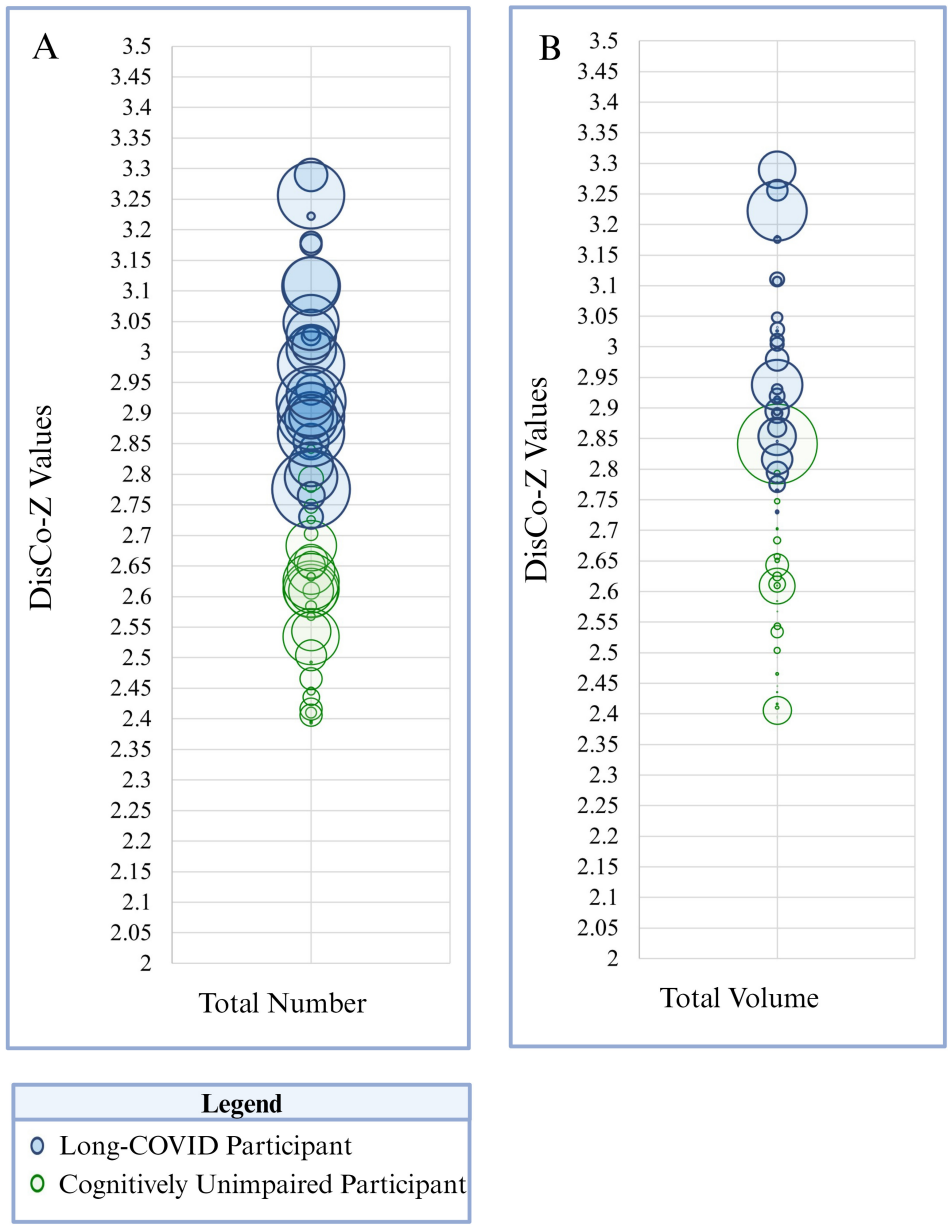
The size and frequency of extreme positive CVR clusters is presented here. **Panel A** depicts total number of clusters identified across the whole brain. **Panel B** reflects total size (i.e., volume) of extreme positive CVR clusters across the whole brain. Each participant is represented as a circle on the graph. Green circles correspond to participants in the cognitively unimpaired group and blue circles correspond to participants in the Long-COVID group. Circle size corresponds to total number of clusters (**Panel A**) or total volume of clusters (**Panel B**) within the whole brain per participant. DisCo-z values are represented along the vertical axis.

**Figure 3:**
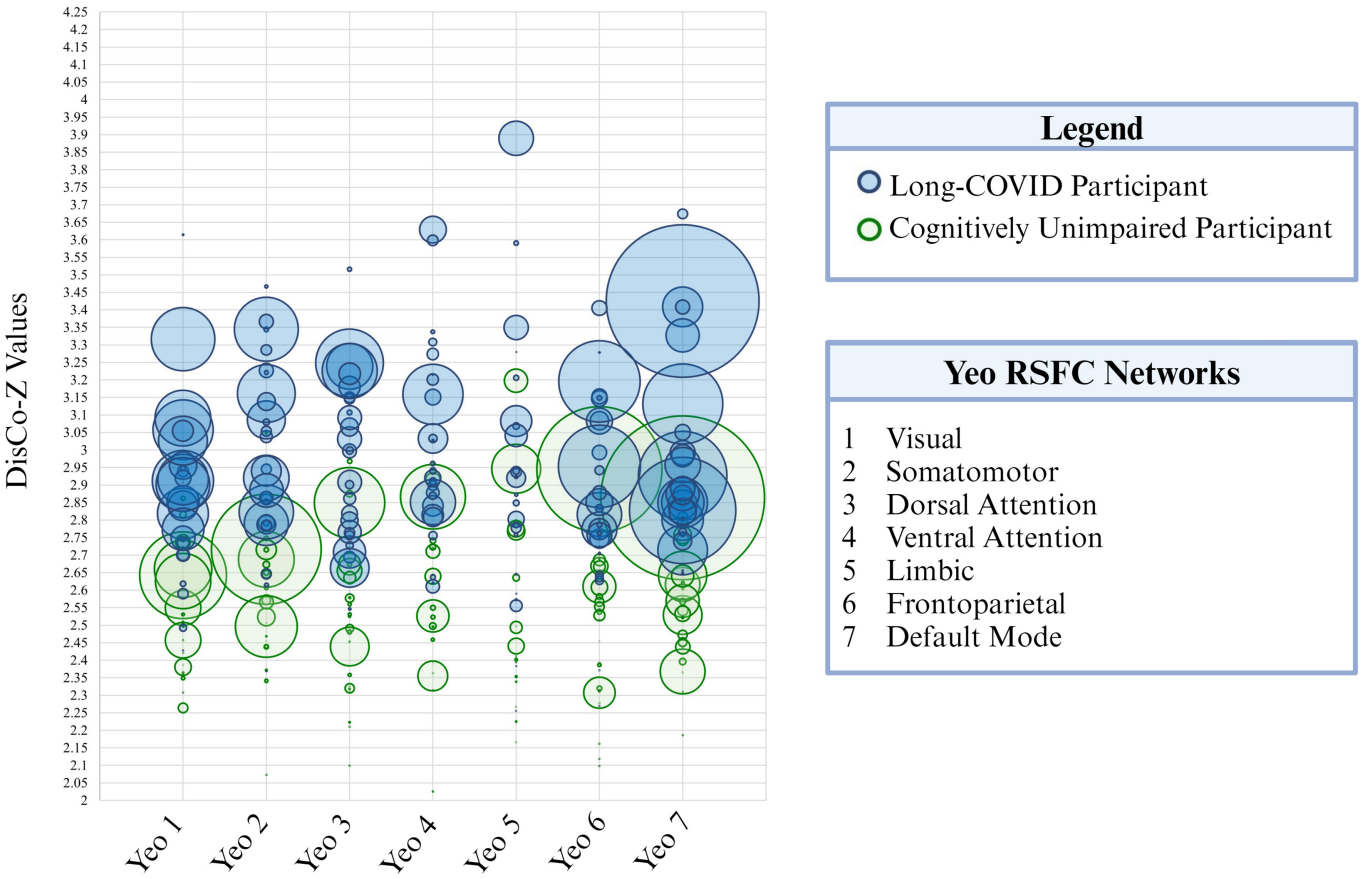
Extreme positive CVR cluster size is presented here for each of the seven Yeo resting-state functional networks. Each participant is represented as a circle on the graph. Green circles correspond to participants in the cognitively unimpaired control group and blue circles correspond to participants in the long-COVID group. Circle size corresponds to cluster size (i.e., total volume). DisCo-Z values are represented along the vertical axis.

**Figure 4:**
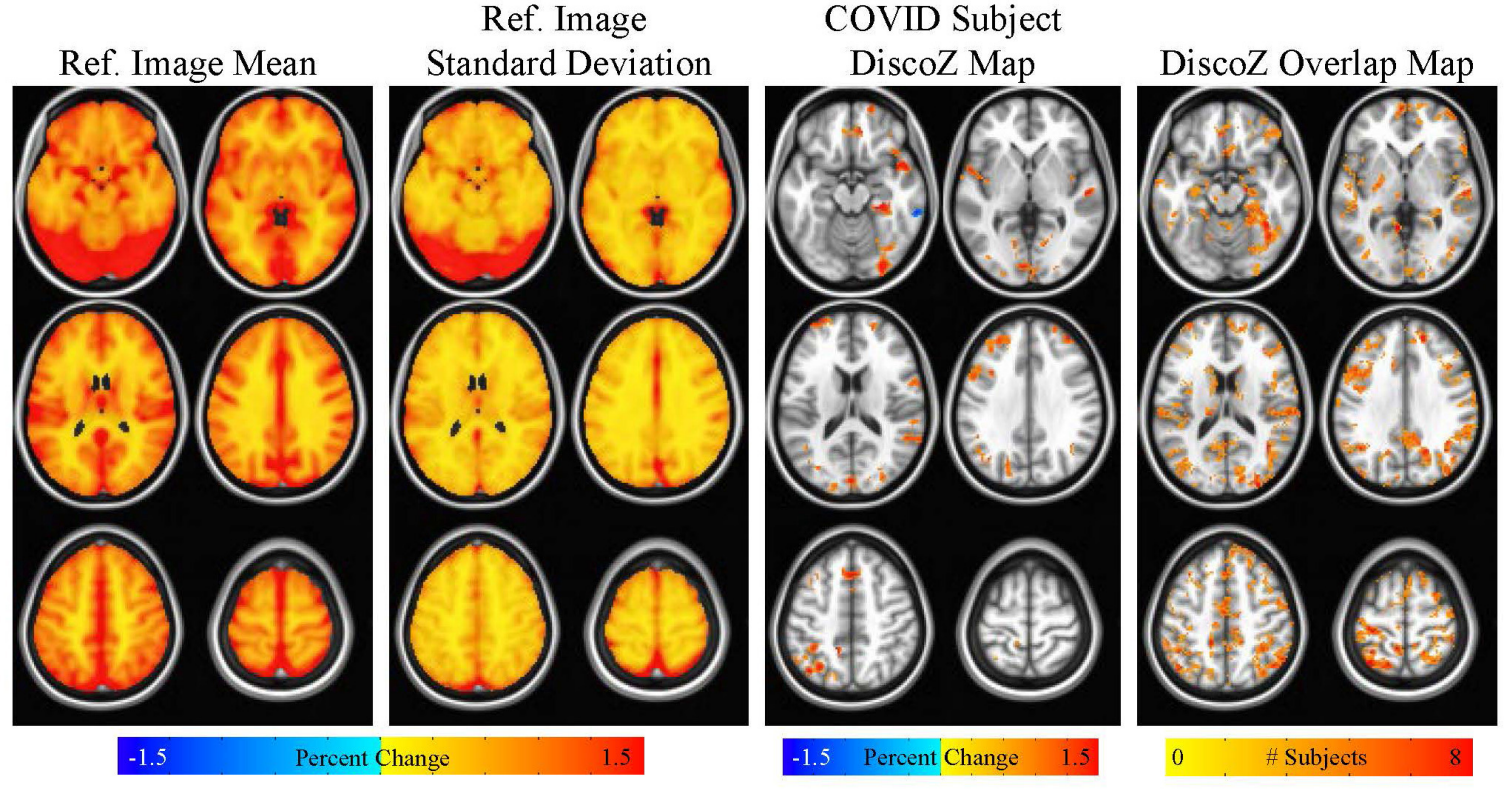
Maps showing the reference CVR mean and reference CVR standard deviation. A DiscoZ map from a representative COVID participant is also shown highlighting extreme positive clusters. Finally, a map showing the number of COVID subjects with extreme positive clusters in each voxel is displayed. This map is thresholded at four subjects (i.e., only voxels with four or more subjects with extreme positive clusters are shown).

Group differences in extreme CVR clusters within the whole brain were examined first. When positive CVR was examined, the long-COVID group demonstrated a significantly higher number of extreme clusters, greater volume of extreme clusters, and higher number of involved networks compared to the control group (corrected *p* = 0.003–0.008). Corrected and uncorrected *p*-values for aforementioned comparisons are presented in Table 3. See Figures 1–4 for graphical representation of CVR and group differences in positive CVR. When negative CVR was examined, the long-COVID group demonstrated a significantly fewer number of extreme clusters and smaller total volume involved (corrected *p* = 0.0036). The long-COVID group had, on average, a greater number of networks that contained extreme positive clusters compared to the control group (corrected *p* = 0.0036). Individual ROIs were examined next.

**Table 3:** Extent and spread of positive and negative extreme CVR clusters >100 Voxels^a^.

	**Extreme positive CVR**			**Extreme negative CVR**		
	**CU**	**LC**	***p*** **-value** ^b^	**CU**	**LC**	***p*** **-value** ^b^
Number of clusters	8.45 (6.50)	14.8 (7.57)	0.002 (0.0036)	0.97 (1.6)	0.13 (0.34)	0.001 (0.0036)
Number of networks	4.45 (2.42)	5.97 (1.91)	0.003 (0.0036)	0.61 (1.05)	0.13 (0.43)	0.008 (0.008)
Total volume	5277.90 (9290.8)	9709.29 (8696.08)	0.002 (0.0036)	194.16 (397.16)	27.9 (76.04)	0.003 (0.0036)

^a^The mean number extreme clusters, the mean number of networks with extreme clusters, and total volume of extreme clusters is presented for each group. CU, cognitively unimpaired; LC, long-COVID; N, sample size; S.D., standard deviation.

^b^*p*-values are presented within the table corresponding to each of the four Mann-Whitney Tests performed. Both the uncorrected *p*-value and corrected *p*-value (in parentheses) is provided for each comparison.

### 3.4 Group differences in extreme CVR clusters: ROI analysis

There were significantly more participants in the long-COVID group that had extreme positive CVR clusters compared to controls for Yeo 2 (Somatomotor), Yeo 4 (Ventral Attention), and Yeo 7 (DMN; *p* = 0.031, *p* = 0.011, and *p* = 0.043, respectively). Mean cluster size was significantly larger for the long-COVID group compared to the control group in Yeo 1 (Visual), Yeo 2 (Somatomotor), and Yeo 3 (Dorsal Attention; *p* = 0.045, *p* = 0.021, *p* < 0.001). Only the dorsal attention network remained significant when corrected for multiple comparisons. See Table 4 for *p*-values.

**Table 4:** Incidence and size of extreme positive CVR clusters (>100 voxels)^a^.

	**Extreme positive CVR incidence** ^**b**^			**Extreme positive CVR size** ^**c**^		
**Region**	**CU**	**LC**	***p*** **-value** ^d^	**CU**	**LC**	***p*** **-value** ^e^
Yeo 1	20/31	27/31	0.073	1327.45	2009.40	0.045 (0.105)
Yeo 2	20/31	28/31	0.031	1301.90	1552.92	0.024 (0.084)
Yeo 3	24/31	27/31	0.508	773.79	1526.25	0.0003 (0.002)
Yeo 4	17/31	27/31	0.011	954.23	1111.51	0.132 (0.154)
Yeo 5	15/31	20/31	0.306	819.20	893.35	0.268 (0.268)
Yeo 6	20/31	27/31	0.073	1165.35	1468.70	0.089 (0.125)
Yeo 7	22/31	29/31	0.043	1755.36	2552.82	0.085 (0.085)

^a^Incidence and size of extreme positive CVR clusters within the long-COVID and cognitively unimpaired samples. CU, cognitively unimpaired; LC, long-COVID; N, sample size; S.D., standard deviation.

^b^Incidence of extreme positive CVR clusters by resting-state network within the long-COVID and cognitively unimpaired samples.

^c^Mean size of extreme positive CVR cluster by resting-state network within the long-COVID and cognitively unimpaired samples.

^d^*p*-values for each Fisher's exact test.

^e^*p*-values for each Mann-Whitney U test.

### 3.5 Clinical correlates of extreme CVR clusters

#### 3.5.1 Self-reported cognitive concerns and extreme CVR clusters

Next, self-reported cognitive symptoms were examined in relation to DisCo-Z values. Within the full sample (*N* = 26), higher total number of self-reported cognitive concerns was positively correlated with DisCo-Z values within 3 of the 7 ROIs [Yeo 2 (Somatomotor): *p* = 0.041, Yeo 4 (Ventral Attention): *p* = 0.001, and Yeo 7 (DMN): *p* =0.016]. When controls were removed from the sample, the total number of self-reported cognitive symptoms was positively correlated with DisCo-Z values for Yeo 4 (Ventral Attention) Network (*p* = 0.034). See Table 5 for *p*-values and correlation coefficients.

**Table 5:** Association between total reported subjective cognitive concerns and Disco-Z metrics^a^.

**Region**	**ρ^b^**	**Significance^c^**	**N^d^**
Yeo 1	0.208	0.307	26
Yeo 2	0.403	0.041	26
Yeo 3	0.384	0.053	26
Yeo 4	0.606	0.001	25
Yeo 5	0.316	0.142	23
Yeo 6	0.249	0.219	26
Yeo 7	0.468	0.016	26
Whole Brain	0.467	0.016	26

^a^The association between subjective cognitive symptoms reported (total number of domains affected) and Disco-Z values for each resting-state network and whole brain.

^b^Spearman rank correlation coefficients.

^c^The *p*-value for each correlation is presented within the significance column. The corresponding sample sizes are presenting in the far right column for each analysis.

^d^Sample size represented for each variable.

#### 3.5.2 Objective cognitive performance and extreme CVR clusters

Within the full sample, objective memory performance was negatively correlated with total number of positive networks with extreme values (Spearman's Rho −0.321; *p* = 0.022) and whole brain total extreme volume (Spearman's Rho −0.315; *p* = 0.029). Trails B was negatively correlated with DisCo-Z values for Yeo 3 (Dorsal Attention); and semantic fluency correlated negatively with Yeo 2 (Somatomotor) values. See Table 6 for *p*-values and correlation coefficients.

**Table 6:** Association between total reported objective cognitive performance and Disco-Z metrics^a^.

**Region**	**Delayed Recall**		**Trails A**		**Trails B**		**Category fluency**		**Letter fluency**		**N^d^**
	ρ^b^	***p*** **-value** ^c^	ρ	***p*** **-value**	ρ	***p*** **-value**	ρ	***p*** **-value**	ρ	***p*** **-value**	
Yeo 1	−0.152	(0.342)	−0.166	(0.299)	−0.108	(0.503)	−0.211	(0.185)	−0.144	(0.370)	41
Yeo 2	−0.305	(0.047)	−0.243	(0.116)	−0.269	(0.082)	−0.433	(0.004)	−0.176	(0.258)	43
Yeo 3	−0.379	(0.009)	−0.135	(0.365)	−0.288	(0.050)	−0.232	(0.117)	−0.140	(0.348)	47
Yeo 4	−0.100	(0.529)	−0.300	(0.054)	−0.300	(0.054)	−0.183	(0.245)	0.041	(0.796)	42
Yeo 5	−0.230	(0.170)	0.036	(0.832)	−0.182	(0.280)	−0.117	(0.489)	−0.123	(0.470)	37
Yeo 6	−0.191	(0.198)	−0.009	(0.952)	−0.103	(0.489)	−0.205	(0.167)	−0.209	(0.158)	47
Yeo 7	−0.236	(0.111)	−0.224	(0.130)	−0.221	(0.136)	−0.190	(0.202)	−0.168	(0.260)	47

^a^The association between neuropsychological performance and Disco-Z values for each resting-state network.

^b^Spearman rank correlation coefficients.

^c^The *p*-value for each correlation is presented within the significance column.

^d^Sample size represented for each variable.

## 4 Discussion

While the mechanisms driving cognitive symptoms in long-COVID are still being explored, older adults, in particular, may be at heightened risk for cognitive decline following COVID-19 infection. Prior studies have highlighted the role of neurovascular health (particularly via changes in endovascular function) as a possible mechanism in long-COVID. Cerebrovascular reactivity, a measure of neurovascular function and endothelial function, has been associated with cognitive changes in older adults. Thus, it reflects a mechanism of particular interest for understanding cognitive changes in an older adult sample with long-COVID. Our study was the first to examine a key marker of neurovascular health, cerebrovascular reactivity, in older adults with long-COVID. We will discuss the key findings of our study, potential clinical implications, and next steps for research, as well as the limitations of our study.

### 4.1 Increased incidence and size of extreme CVR clusters in long-COVID

Our study demonstrated a statistically significant increase in presence of extreme CVR clusters in the long-COVID group. Extreme CVR clusters occurred at a greater frequency within the long-COVID group when the whole brain was examined and within each of the seven resting-state networks. Similarly, the mean size of extreme CVR clusters was significantly larger within the long-COVID group when whole brain was examined and within resting-networks. CVR has previously been conceptualized as a “brain stress test.” Potentially, the increase in size and incidence of extreme CVR clusters could be conceptualized as a proxy for the overall responsiveness of the cerebrovascular system. Extreme positive or negative CVR values could suggest a more dysregulated neurovascular response. While there is relatively less literature, in general, on the clinical significance of increased CVR, one hypothesis that has been discussed in the literature to explain increased CVR is the steal phenomena, whereby lower extreme values suggest less responsiveness in a given region. While the purpose of this investigation was not to assess the steal phenomenon, future studies might examine this as a possible factor. Potentially, these findings reflect the neuroinflammatory and vascular changes previously observed as a driving mechanism of long-COVID in other studies (56). Alternatively, these findings could reflect a premorbid group difference that places individuals at heightened risk for developing long-COVID. Additional studies are needed to better disentangle the directionality of these findings.

### 4.2 Higher number of subjective cognitive symptoms in long-COVID associated with extreme CVR clusters in ventral attention network

Within the long-COVID sample, the total number of subjective cognitive symptoms reported was significantly positively correlated with presence of CVR extreme values within the ventral attention network. Prior studies in long-COVID have differentiated the subjective cognitive symptoms from objective cognitive changes on neuropsychological measures suggesting different possible mechanisms and time course. Our findings suggest that the experience of subjective cognitive changes in long-COVID may be linked to neurovascular function in attentional networks. While further research is needed to disentangle these findings, potentially, individuals the experience of subjective cognitive changes could reflect less efficient attentional networks. Given prior work that has highlighted changes in functional attentional networks with age, additional research examining longitudinal changes in attentional networks in the context of long-COVID would be of interest.

Age-related physiological changes in neurovascular function in older adults have been hypothesized to place older adults at greater risk for development of long-COVID. In particular, endothelial dysfunction, which has been characterized as a hallmark of age-related vascular decline (57) has been identified as a mechanism of interest in long-COVID (58, 59) as well. Briefly, endothelial cells form the lining of blood vessels and serve a variety of different functions necessary for maintaining vascular health and play a key role in oxidative stress, neuroinflammation, and innate immunity. Research has suggested endothelial cells are particularly vulnerable to COVID-19 and disruption of endothelial function (e.g., via increased oxidative stress, reduction in the bioavailability of nitric oxide, etc.), may drive the continued symptoms in long-COVID (60).

### 4.3 Greater incidence and size of positive extreme CVR clusters associated with worse objective memory performance in older adults

Prior studies have linked declines in CVR to worse objective memory performance in older adult sample. Notably, those studies were examining a clinical decline or change in objective memory scores (e.g., in context of mild cognitive impairment or when comparing young adults to older adults). The participants within this study demonstrated average or better memory scores based on normative samples. Within our study, global measures of CVR burden were associated with worse verbal memory performance. Our study is the first to highlight the relationship between extreme CVR clusters and objective memory performance. Potentially, one could hypothesize that subtle neurovascular changes precede more overt declines in CVR that have previously been linked associated to memory decline. Finally, our findings raise the possibility that subtle neurovascular changes (evinced by more extreme CVR clusters) could reflect a pathway by which COVID-19 theoretically could accelerate age-related declines in memory.

### 4.4 Further support for utility of distribution-corrected z-score approach

Prior studies have demonstrated the utility of a subject-specific approach for examining neuroimaging changes in clinically heterogenous disease states, such as Multiple Sclerosis and Traumatic Brain Injury (34–38). Our study is the first to adopt this approach in a long-COVID sample. Further, while SSA including DisCo-Z have been used when examining functional connectivity and DTI, our study is the first to demonstrate the utility of this approach in the study of cerebrovascular reactivity. Overall, these findings provide further support for this statistical approach broadly, and highlight its value in furthering the field's understanding of both long-COVID and CVR.

### 4.5 Limitations

There a several limitations to consider when interpreting the findings of the present study. We recruited older adults already receiving care for long-COVID, which may reflect a more severe sample relative to the general population. Further, we recruited individuals who specifically were endorsing subjective cognitive symptoms associated with COVID-19 infection. Perceived cognitive changes is a construct studied in other neurological conditions (e.g., mild Traumatic Brain Injury, Mild Cognitive Impairment). Potentially, a systemic bias may be introduced when targeting this cohort that could be addressed in future studies with inclusion of additional comparison groups (e.g., individuals with subjective cognitive concerns without a history of COVID-19, individuals with long-COVID that are reporting only physical symptoms). Given the heterogeneity in COVID-19 variants with different exposure to vaccine (as some participants were acutely infected with COVID-19 prior to development of vaccines), future studies with larger sample sizes could examine the role of variants and additional covariates could be examined and controlled for statistically [e.g., medications, comorbid vascular health conditions (including hypertension, diabetes, hyperlipidemia), total number of infections, timing of cognitive symptoms in relation to vaccination]. Similarly, as our study was conducted at a single time point (after development of long-COVID) we cannot determine whether the group differences reflect a preexisting condition that increases risk for long-COVID. Data was combined from two separate studies and neuropsychological test scores were only obtained from a subset of individuals for whom a clinical neuropsychological evaluation had been completed as part of standard of care. Consequently, there was some variability in the specific tests used relative to the control group (all of whom received a standard battery) and who completed the cognitive symptom questionnaire. Additionally, there are limitations to self-report measures of cognition. Future studies would benefit from additional sources of data to establish presence of observed cognitive change (e.g., collateral report) as well as use of normed behavioral questionnaires around subjective cognitive change. Regarding demographic make-up, the present sample was a predominantly non-Hispanic, White sample which limits the extent to which findings can be generalized to the general population. Additionally, the long-COVID sample was younger than the control sample, though we would hypothesize this group difference would be more likely to minimize the group difference rather than explain the difference. To better assess this however we examined the correlation between the variables of interest (extreme CVR clusters) and age and did not find a statistically significant relationship. Finally, the current study recruited older adults who reported no cognitive concerns of any kind but did not explicitly assess for COVID infection history. Given the prevalence of COVID-19, heterogeneity of strains and immunity over time, it would be challenging, but ideally, a third control group would be included comprised of older adults who had contracted COVID-19 and reported no changes in cognition. Further, the present sample was comprised predominantly of female participants. This is a reflection of the sample collected. While there has been some research that has suggested a greater reported of cognitive symptoms in women relative to men with long-COVID, in the context of this study, it could also reflect openness to research participation more broadly. Given the relatively small sample size, we do not have the power to examine the independent effect of sex as it relates to long-COVID, however, we did match participants based on sex and we have regressed out the effects of sex when appropriate (e.g., use of normative reference groups for neuropsychological data that consider sex, statistically controlling for sex in CVR analyses). Future studies examining sex more directly are of interest for understanding long-COVID, though unfortunately with the current sample size this could not be explored. Finally, regarding CVR, there are limitations specific to the breath holding task. Efforts were made to address limitations in the following ways: participants were instructed to perform BH on expiration only which has been shown to be more repeatable than BH on inspiration, a paced breathing paradigm was used to control participants' breathing rate, and finally, respiratory traces were collected for all participants and manually inspected to ensure each participant performed 4 breath holds. Future studies controlling for end tidal pressure CO_2_ would be recommended.

## 5 Conclusions and future directions

The results from this study suggest older adults with long-COVID exhibit alterations in cerebrovascular reactivity compared to cognitively unimpaired older adult sample. In particular, more extreme CVR values were observed in the long-COVID group which were also associated with a greater number of total subjective cognitive symptoms. While acute management of COVID-19 infection has drastically improved, a significant proportion of individuals report prolonged symptoms in the months following resolution of acute COVID-19 infection. Potentially, CVR could be examined over the course of long-COVID or examined as a risk factor for development of long-COVID. CVR has been hypothesized as a potential target for treatment (61) and could be a target of interest for Long-COVID. While long-COVID is a relatively new syndrome, there is a larger body of literature on cognitive changes in the other post-infectious disease states. Our findings may have utility for the analysis of other post-infectious states associated with cognitive changes (e.g., Myalgic encephalomyelitis/chronic fatigue syndrome, Lyme Disease, etc) as well.

Long-COVID encompasses a wide range of symptoms that must be understood within both the context of an individual's health history and the broader dynamics of the ongoing pandemic, including variations in viral strains, vaccine timelines, and other evolving factors. This study focused on persistent cognitive changes among older adults, but these symptoms exist alongside other manifestations such as dyspnea, palpitations, peripheral neuropathy, and psychiatric changes (e.g., anxiety). Moreover, the emergence and progression of long-COVID symptoms show different patterns over time depending on the aspect of health being assessed. Early autonomic nervous system (ANS) changes, such as alterations in heart rate variability, typically linked to fatigue, may resolve within 6 to 13 months post-infection (62). In contrast, cognitive symptoms can persist for a longer duration, from 6 to 113 months post-infection. Notably, in that study presence of cognitive symptoms was not correlated with ANS functioning, suggesting that mechanisms such as neuroinflammation or microvascular dysfunction may underlie prolonged cognitive concerns. Previous studies have also highlighted the role of vascular risk factors (particularly hypertension, but also cardiovascular disease or diabetes) as well as older age (63), prior infections or vaccine exposure in modulating long-COVID outcomes and immune responses to vaccination (64). Aforementioned comorbid conditions also have been found to have an impact on CVR, as well as cognition in older adulthood, again highlighting the needed for future studies that can examine these complex processes.

There is emerging evidence suggesting that long-COVID may impact physiological processes associated with biological aging more directly (e.g., via inflammation and oxidative stress) (65). This could provide a useful framework for understanding the final finding of this study, where more extreme cerebrovascular reactivity (CVR) values were associated with worse objective memory performance. While reduced CVR is linked to cognitive decline in both pathological and normal aging, a DisCo-Z approach to CVR may capture more subtle changes in neurovascular function that affect memory in older adults. In the broader context of long-COVID, one study found evidence of accelerated biological aging in individuals with acutely asymptomatic or mild COVID-19 infection (65). Specifically, 1-year post-infection, these individuals exhibited increased DNA methylation age (DNAmAge) and shortened telomere length (TL) (65). This acceleration in biological aging could potentially explain the cognitive symptoms observed in older adults with long-COVID, and might also help to explain the broader relationship between CVR and memory in the full sample. Future studies should explore this mechanism further to better understand its relationship to cognitive impairment in long-COVID. Additionally, persistence of the SARS-CoV-2 spike protein has been observed in brain samples and meninges following resolution of acute infection (66). The authors demonstrated that the persistence of the spike protein was associated with chronic inflammation and biomarkers associated with neurodegeneration. Future exploration of the spike protein as a mechanism associated with both cognitive symptoms in long-COVID and CVR would be of interest.

## Data Availability

The datasets presented in this article are not readily available because per local Institutional Review Board regulations, data presented in this publication shall be made available upon formal written request to the authors. Access is contingent on execution of a legally binding data-sharing agreement, signed by both parties, governing the use, protection, and dissemination of shared data. Of note, as data collection is ongoing, the data-sharing agreement would be contingent upon data collection completion and closure of study project. Requests to access the datasets should be directed to yangwang@mcw.edu.
