# Supplementary material for: Changes in cerebrovascular reactivity within functional networks in older adults with long-COVID
Source: Front Neurol. 2025 Mar 26;16:1504573. doi: 10.3389/fneur.2025.1504573 (PMC11981175; doi:10.3389/fneur.2025.1504573)
Supplement: Supplementary file 1 [file Table_1.docx]

Supplemental Table 1. Associations between Age and Extreme CVR Clusters (>100 voxels)^a^.

| CVR Variable | ρ^b^ | Significance^c^ | N^d^ |
| --- | --- | --- | --- |
| Total Extreme Negative CVR Clusters | -.094 | 0.468 | 62 |
| Extreme Positive CVR Clusters | .324 | 0.010 | 62 |

^a^The association between age and total number of extreme CVR clusters within the whole brain.

^b^Spearman rank correlation coefficients.

^c^The p-value for each correlation is presented within the significance column.

^d^Sample size represented for each variable.

Supplemental Table 2. Incidence and size of extreme negative CVR clusters (>100 voxels)^a^.

| **Region** | **Extreme Negative CVR Incidence^b^** | | | **Extreme Negative CVR Size^c^** | | |
| --- | --- | --- | --- | --- | --- | --- |
|  | **CU** | **LC** | **p-value^d^** | **CU** | **LC** | **p-value^e^** |
| Yeo 1 | 2/31 | 0/31 | 0.492 | 449.50 |  | . |
| Yeo 2 | 1/31 | 0/31 | 1.0 | 153.00 |  | . |
| Yeo 3 | 2/31 | 0/31 | 0.492 | 124.50 |  | . |
| Yeo 4 | 2/31 | 0/31 | 0.492 | 108.50 |  | . |
| Yeo 5 | 2/31 | 1/31 | 1.0 | 546.00 | 171.00 | .667 |
| Yeo 6 | 3/21 | 0/31 | .238 | 228.66 |  | . |
| Yeo 7 | 7/31 | 3/31 | .301 | 234.14 | 119.66 | .517 |

^a^Incidence and size of extreme negative CVR clusters within the long-COVID and cognitively unimpaired samples. CU, cognitively unimpaired; LC, long-COVID; N, sample size; S.D., standard deviation.

^b^Incidence of extreme positive CVR clusters by resting-state network within the long-COVID and cognitively unimpaired samples.

^c^Mean size of extreme positive CVR cluster by resting-state network within the long-COVID and cognitively unimpaired samples.

^d^p-values for each Fisher’s exact test.

^e^p-values for each Mann-Whitney U test.
